# Food, mammals, & countries: Stimuli, their pairwise similarities, and MDS dimension values for modeling judgment, categorization, and memory in three real-world domains

**DOI:** 10.3758/s13428-026-03114-w

**Published:** 2026-08-12

**Authors:** David Izydorczyk, Arndt Bröder

**Affiliations:** Department of Psychology, School of Social Sciences, University of Mannheim, Mannheim, Germany

**Keywords:** Cognitive psychology, Similarity, Multidimensional scaling, Experimental stimuli, Categorization, Judgment, Decision-making

## Abstract

Three comprehensive sets of naturalistic stimuli, each with pairwise similarity ratings and multidimensional scaling (MDS)-based feature representations are presented, freely available on the corresponding OSF repository of this project under a CC-BY-SA-NC-4.0 license. The primary contribution of this work is the provision of these well-curated naturalistic stimulus sets and their associated high-quality similarity data as a resource for the research community. The sets include 80 representative items from three domains – foods, mammals, and countries – that can be used across cognitive research areas such as categorization, multiple-cue probability learning, judgment, decision-making, memory, and metamemory. Based on over 280,000 similarity judgments from N=1798 participants, we derived 14 dimensions for foods, ten for mammals, and ten for countries, which reconstructed the similarity spaces with high accuracy (r≥.93). In a proof-of-concept study, these dimensions also explained substantial variance in participants’ numerical judgments of domain-specific criteria, underscoring their functional relevance beyond similarity tasks. By making these stimuli, similarity data, and derived feature spaces freely available, we provide researchers with tools for testing computational cognitive models in relevant real-world domains, each of which allows for examining a multitude of judgment or classification criteria.

**Supplementary Information:**

The online version contains supplementary material available at https://doi.org/10.3758/s13428-026-03114-w.

## Introduction

A central goal of cognitive psychology is to understand how people make decisions, categorize objects, estimate values, and remember information. Over the last decades, many theories of these higher-order processes have reached a high level of formalization, often being implemented as computational models (Farrell & Lewandowsky, 2018; Sun, 2008). Across domains, these models typically assume that stimuli are represented in terms of identifiable *cues*, *attributes*, *dimensions*, or *features* (Bower, 1967; Estes, 1950; Shiffrin & Steyvers, 1997; Tversky, 1977)^1^. This representation of stimuli as a set of cues is crucial because formal models take these features as input for their computations (Brehmer, 1994; Juslin, Olsson, & Olsson, 2003; Medin & Schaffer, 1978; Nosofsky, 1984).

However, because real-world stimuli – such as images of birds, foods, or consumer goods – rarely come with predefined cue structures, researchers typically have relied on simple artificial stimuli whose features can be precisely defined and experimentally controlled. Examples include geometric shapes (e.g., Seitz, Albrecht, Von Helversen, Rieskamp, & Rosner, 2025; Shepard, Hovland, & Jenkins, 1961), fictitious bugs varying on binary features (e.g., Juslin, Karlsson, & Olsson, 2008; Trippas & Pachur, 2019), or comic figures (e.g., Bröder, Undorf, & Gututui, 2025; Hoffmann, von Helversen, & Rieskamp, 2014). While these stimuli allow for rigorous model testing, the extent to which these computational models developed and tested with these stimuli generalize to real-world cognitive phenomena has received comparatively little empirical attention. In particular, the reliance on artificial stimuli raises questions about external validity: How well do results obtained under such conditions generalize to the complex objects and environments in which cognition naturally occurs (Goldstein & Hogarth, 1997)?

We address this gap by providing researchers with three naturalistic stimulus sets from different domains – foods, mammals, and countries – along with their pairwise similarity ratings and derived feature representations based on multidimensional scaling (MDS; Borg & Groenen, 2005; Kruskal, 1964; Shepard, 1962a). These MDS-based features can serve as inputs for cognitive models, enabling theory-driven research on memory, categorization, judgments, and decisions with realistic materials across a broad range of cognitive tasks. The primary contribution of the present work is therefore the provision of a well-curated database of naturalistic stimuli, including pairwise similarity ratings and derived representational spaces.

In the remainder of this article, we will first outline the motivation for providing the stimulus sets, their pairwise similarity ratings, and derived feature representations by reviewing the stimuli used in previous approaches. Next, we will describe the selected domains and the stimulus sets. In the second part of the article, we report on similarity ratings and their MDS analysis that resulted in feature representations of the stimuli in each domain. Finally, in a proof-of-concept study, we asked additional participants to judge a target dimension in each domain after a brief training phase and assessed the relevance of the extracted dimensions by examining how much variance they explained in these judgments when used as predictors.

### Stimuli and cues used in former approaches

Model-based research in memory, categorization, judgment, and related cognitive domains has employed a wide range of stimulus materials that differ in a) the realism of the stimuli and b) how the properties used to represent these stimuli are generated. Throughout this section, we use the term cues (or features/dimensions, see Footnote 1) to refer to constituting and observable or inferred properties of stimuli that serve as inputs to cognitive models. A dimension, therefore, denotes a variable along which stimuli can vary (e.g., size, color, or presence of an airport), whether experimentally defined or derived from data.

In most studies, researchers define or hand-code the cues used to represent stimuli themselves. However, the stimuli derived from these cues can differ significantly in their realism, which may influence how well findings generalize beyond laboratory settings. Accordingly, prior work can be arranged along a continuum of stimulus realism, ranging from highly artificial materials to naturalistic real-world stimuli.

At the most abstract end of this continuum are highly artificial stimuli, such as geometric shapes that vary along predefined attributes like shape, size, length, numerosity, or color (e.g., Ashby & Maddox, 1990; Bruner, Goodnow, & Austin, 2017; Donkin, Newell, Kalish, Dunn, & Nosofsky, 2015; Goldstone, 1994; Medin & Schaffer, 1978; Seitz et al., 2025). These stimuli have the clear advantage of minimal pre-existing semantic associations, making them largely independent of prior knowledge. While they allow for flexible definitions of category structures and cue-criterion relations in a blank-slate learning perspective, they lack direct relevance or face validity for everyday tasks. Importantly, we do not devalue controlled artificial stimuli as a means of theory testing. Such simplified laboratory tasks provide an essential foundation for isolating mechanisms and deriving precise predictions. However, cumulative theoretical progress requires demonstrating that models generalize beyond tightly controlled settings, as psychology ultimately seeks to explain and predict behavior in real-world environments (Brunswik, 1955).

In the attempt to approximate more realistic domains while retaining control over stimulus structure, researchers have used quasi- or semi-realistic stimuli. These stimuli maintain the combinatorial cue structure of artificial materials but add semantic content. Examples include "death bugs" introduced by Juslin et al. (2003, see Bergert and Nosofsky, 2007; Juslin et al., 2008; Nosofsky and Bergert, 2007; Trippas and Pachur, 2019), hypothetical stocks (e.g., Bröder, 2000; Newell & Shanks, 2003; Nilsson, Olsson, & Juslin, 2005), hypothetical diseases predicted by symptoms (e.g., Bröder, Newell, & Platzer, 2010; Gluck & Bower, 1988; Persson & Rieskamp, 2009), job candidates (e.g., Rosner & von Helversen, 2019; von Helversen, Karlsson, Rasch, & Rieskamp, 2014), fictitious flowers or butterflies (e.g., Hoffmann, von Helversen, & Rieskamp, 2016; Izydorczyk & Bröder, 2022), fictitious murder suspects (Bröder & Schiffer, 2003), or cartoon characters (Bröder et al., 2025; Hoffmann et al., 2016, 2014)^2^. Although these stimuli allow for various forms of semantic enrichment, their construction from a set of predefined basic features still provides a high degree of experimental control and ensures that the features are relevant to the tasks.

In the Brunswikian tradition of judgment research, human judgments are conceptualized within the framework of the lens model (Brunswik, 1952, 1955; Karelaia & Hogarth, 2008), which assumes that people infer a criterion variable through probabilistic cues available in the environment. Judgment accuracy depends both on how strongly cues are related to the criterion (the ecological structure) and on how well individuals utilize these cues in forming their judgments (Cooksey, 1996; Hammond, 1955). Within this framework, several studies have used naturalistic stimuli, such as cities (Gigerenzer & Goldstein, 1996), medical (Laduca, Engel, & Chovan, 1988) or forensic cue profiles (Cooper & Werner, 1990; Werner, Rose, & Yesavage, 1990), student descriptions (Cooksey & Freebody, 1986; Cooksey, Freebody, & Wyatt-Smith, 2007), or food items (Bröder, Dülz, Heidecke, Wehler, & Weimann, 2023), and then used cues that the researchers deemed relevant or cognitively plausible for explaining how people make their judgments. For instance, in their city-size task, Gigerenzer and Goldstein (1996) defined potentially relevant (binary) probabilistic cues, such as the presence of an airport or a major league soccer club. However, this cue selection is guided by the researcher’s intuition, and it remains unclear whether these post hoc cues are indeed functionally relevant.

To overcome the limitations of researcher-designed and hand-coded cues, an alternative is to derive features directly from data. One approach is to use similarity judgments to construct multidimensional scaling (MDS) representations, in which stimuli are represented as points in a multidimensional space, so that similar stimuli are located closer together than dissimilar stimuli (Hout, Papesh, & Goldinger, 2013; Shepard, 1962a). For example, Shin, Nosofsky, and UniversityBloomington (1992) applied this approach to artificially generated dot patterns, deriving MDS-based features that were subsequently used as inputs for cognitive models of classification and recognition. Similarly, Cooke, Jäkel, Wallraven, and Bülthoff (2007) demonstrated the method with novel three-dimensional objects explored visually and haptically, showing that MDS representations can capture shared structure across sensory modalities and support categorization modeling. These studies illustrate how similarity-based feature extraction provides a principled, data-driven way to generate model inputs from controlled perceptual stimuli.

Extending this data-driven approach beyond artificial stimuli, Storms and colleagues used similarity judgments and MDS-derived representations within exemplar and prototype models to predict phenomena such as typicality and categorization in natural language categories (Ameel & Storms, 2006; Smits, Storms, Rosseel, & De Boeck, 2002; Storms, De Boeck, & Ruts, 2001; Voorspoels, Vanpaemel, & Storms, 2008). For instance, Smits et al. (2002) applied multidimensional scaling to similarity judgments for natural language categories and concepts, deriving representational dimensions for familiar and novel food items (fruits and vegetables) and using these MDS-based representations as inputs for competing categorization models. Building on this demonstration that similarity-derived representations can capture structure in meaningful real-world categories, Nosofsky and colleagues later developed a systematic program aimed at deriving functionally relevant features (i.e., features which are relevant for participants’ cognitive processing in a given task) for complex naturalistic stimuli (R. M. Nosofsky, Sanders, & McDaniel, 2018a, b; R. M. Nosofsky, Sanders, Meagher, & Douglas, 2018c, 2020; R. M. Nosofsky, Sanders, Zhu, & McDaniel, 2019). Using similarity judgments for large sets of rocks and minerals from geologically defined classes, they constructed MDS-based representational spaces and showed that the resulting dimensions could successfully serve as inputs for computational categorization models and guide the selection of optimal training exemplars for category learning (Nosofsky et al., 2018a, b; Nosofsky et al., 2018c; R. M. Nosofsky et al., 2019) and also recognition-memory tasks (R. M. Nosofsky, Ehinger, & Osth, 2025; R. M. Nosofsky & Osth, 2026). More recently, Izydorczyk and Bröder (2024) adapted this method to an estimation task in which participants estimated the actual maximal flight speed of 32 bird species after receiving feedback about the direct (e.g., "This bird has a max. flight speed of 34 km/h") or relative (e.g., "Bird A is faster than Bird B") flight speeds of 12 of these birds during a previous training phase. They derived MDS-based features for all 32 birds and used these features as inputs for a computational model of the estimation process. By doing so, they replicated a key finding of previous experiments with artificial stimuli (Pachur & Olsson, 2012; Trippas & Pachur, 2019), showing that the type of learning and feedback during the estimation task influences the underlying estimation process. Thus, by deriving functionally relevant cues directly from similarity judgments, the MDS approach overcomes the limitations of researcher-defined features, providing a data-driven method to study real-world stimuli and bridge the gap between ecological validity and experimental control.

Instead of deriving features from human similarity judgments via MDS, more recent studies use deep neural networks (DNNs) to generate high-dimensional stimulus representations. In this approach, researchers pass images or words through a pre-trained network, often optimized for object recognition or language modeling, and extract the resulting feature vectors. These vectors can then be used as inputs to cognitive models to predict human judgments and behavior (e.g., Aka, Bhatia, & McCoy, 2023; Bhatia & Stewart, 2018; Jha, Peterson, & Griffiths, 2023; Peterson et al., 2016; Richie, Zou, & Bhatia, 2019). For example, Battleday, Peterson, and Griffiths (2020) used image embeddings from popular vision models as stimulus representations to model participants’ categorizations of naturalistic and complex stimuli. Compared to MDS, DNN-based representations can be generated quickly and at minimal cost for vast numbers of stimuli, without collecting similarity ratings from participants. However, this efficiency comes with a cost: features extracted from DNNs tend to reflect the network architecture and the specific task used during training. Network representations become progressively more task-specific in higher layers, and their transferability to other tasks decreases as task differences grow (Jang, Plis, Calhoun, & Lee, 2017; Yosinski, Clune, Bengio, & Lipson, 2014). Human similarity ratings, in contrast, directly reflect the rich mental representations of individual participants.

Therefore, building on the MDS approach by Nosofsky and colleagues, this article provides researchers with stimulus sets from three real-world domains, including their pairwise similarity ratings and MDS-based features. Our goal is not to introduce new methodologies for multidimensional scaling or feature extraction; rather, this paper serves as a resource-oriented contribution. We provide the research community with a well-curated database of large sets of naturalistic stimuli, with corresponding similarity ratings and features derived by non-metric MDS. These resources are intended to further foster the application of computational cognitive models to realistic stimuli. In the next sections, we will introduce the domains and the actual stimulus sets.

## The domains

Building on the pioneering work of Nosofsky and colleagues, we aimed to select stimuli from varying domains that would be more broadly applicable than the minerals domain. We eventually chose the domains of food items, animals (mammals in particular), and countries based on three criteria.

First, we searched for domains with high everyday relevance. Although minerals and rocks are a good example of a scientifically defined category, these stimuli can be used primarily for novices (who are just starting to learn about rocks) or for experts. In contrast, the domains introduced here are expected to be both everyday-relevant and to require some expertise, even among laypeople. This everyday relevance may also help maintain participant motivation in laboratory or online settings.

Second, the domains should be suitable for a large variety of potential judgment or decision dimensions. For example, food items can be judged on attributes like caloric content, carbon footprint, sugar content, and many other criteria. Similarly, countries can be evaluated by metrics such as population, gross national product, and life expectancy, among other criteria. Hence, the domains are flexible with respect to the judgment or categorization task used in studies. This also allows for addressing very different cognitive representations and degrees of difficulty in the tasks.

Third, we strove for one natural taxonomic domain (mammals), a purely cultural/political domain (countries), and one mixed domain (food items). The food domain was also chosen because of its high relevance for public health, as nutritional literacy is critical for both the general population and specific patient groups (e.g., diabetics). The inclusion of this domain can therefore directly benefit basic research aimed at optimizing judgments in a highly relevant domain.

While large-scale resources like the THINGS database (a multimodal collection of neuroimaging and behavioral datasets for over 1850 object concepts; M. N. Hebart et al., 2019; M. N. Hebart et al., 2023; Stoinski, Perkuhn, & Hebart, 2023) aim for maximal coverage over a large range of different objects, our goal was to provide a large, representative set of items within each domain. This approach allows researchers to investigate subtle within-domain differences in behavioral tasks that would be difficult to capture in a breadth-oriented database with only a few exemplars per concept. We therefore selected 80 representative items for each domain (see next section). This number was chosen to allow for a reasonably large set to select from in experiments while keeping the demand for similarity judgments for MDS analysis manageable (see below).

## Stimulus sets

**Fig. 1:**
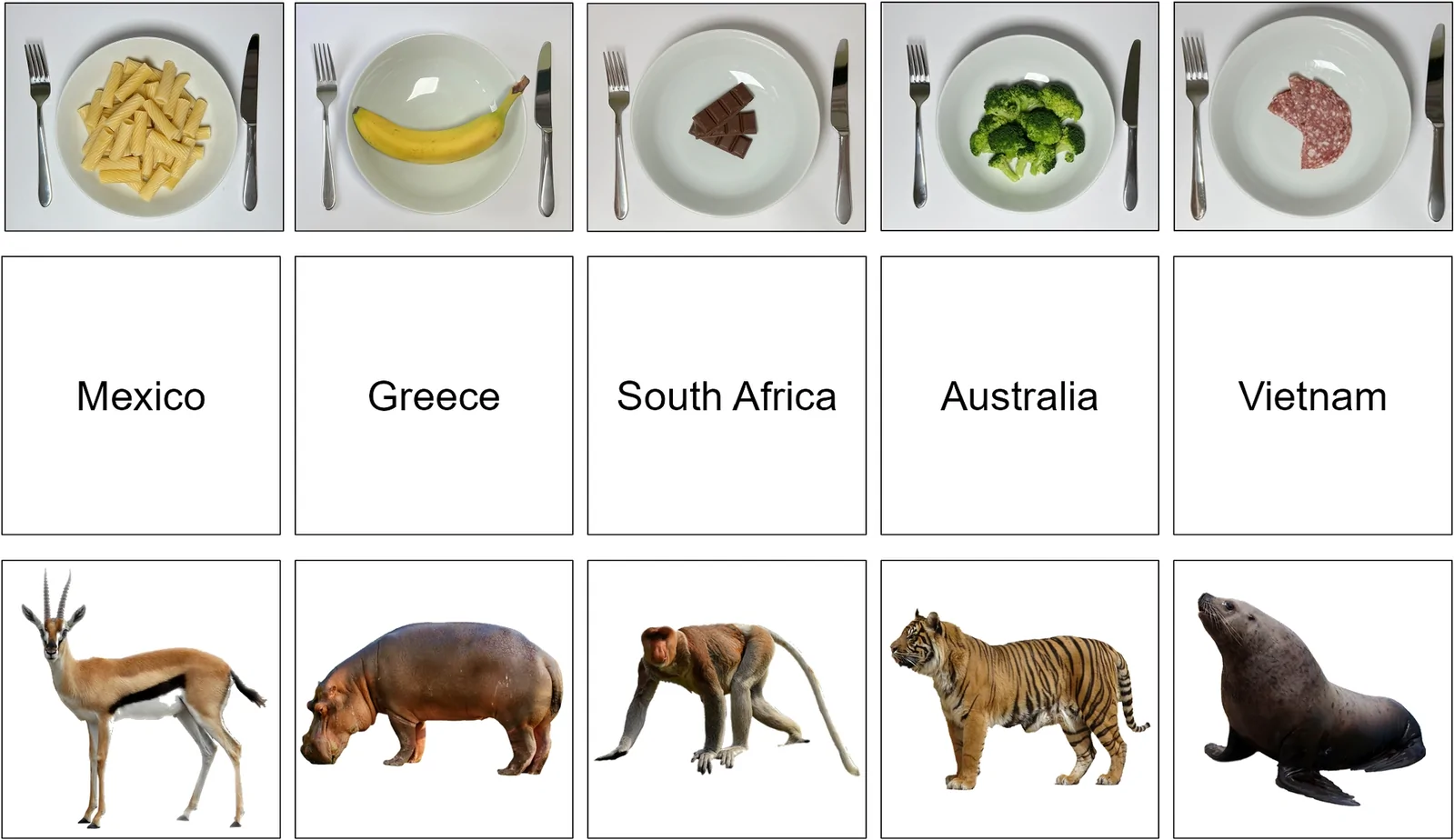
Example stimuli of each domain

We selected 80 representative stimuli for each of the three domains to ensure broad coverage of the respective stimulus space while maintaining a set size that is reasonably large for experimental validity yet still manageable for the pairwise similarity rating task (details below). Figure 1 shows five examples for each domain, while the complete lists of stimuli are provided in Tables 2 to  4.

Although we tried to cover a broad range of different stimuli within each domain, we also included several pairs of very similar items, which might prove useful for a specific cognitive task or research questions. Details for all stimuli and the corresponding images can be found at the OSF, where all images are freely available for use in non-commercial research under a CC-BY-SA-NC-4.0 license.

### Food

For the food domain, we first compiled a list of food items commonly consumed by members of our lab, students, and acquaintances. From this pool, we selected 80 frequently mentioned items representing a broad range of typical food categories, including fruits, vegetables, meats, and sweets. Items include common staple foods such as rice, apples, bell peppers, and chicken, as well as more processed items like cherry jam, chips, and cheesecake. All photographs were taken with an iPhone 13 Pro under standardized conditions: each food item was presented on the same white plate, with a fork and knife placed beside it for scale and context.

### Countries

Using the list of countries and dependencies provided on Wikipedia (List of countries and dependencies, 2024), we randomly sampled 80 countries while ensuring balanced numbers of countries across six continental regions: Africa, Asia, South America, North and Central America, Europe, and Oceania. We aimed to create a set that could support judgments, categorizations, or decisions on multiple types of attributes, for example, economic indicators (e.g., GDP), demographic measures (e.g., population, life expectancy), or cultural factors (e.g., language diversity).

### Mammals

For the mammal domain, we selected 80 species spanning a wide range of taxonomic categories, families, and genera, differing in size, habitat, body shape, and nutrition. Examples included the guinea pig, African elephant, llama, bottlenosed dolphin, and gray wolf. Images for each species were chosen to present the animal in a clear side view, showing the full body, and were available under appropriate licenses. To ensure visual consistency, all images were edited using Adobe Photoshop (Version 26): each animal was cropped from its original background and placed on a uniform white background to ensure visual consistency. A complete list of image sources and authors is available at OSF.

Thus, across all three domains, our design choices aimed to balance experimental control and representativeness of the respective domain. By combining taxonomic, conceptual, and mixed domains, and by ensuring that each set offers both broad variation and close-item pairs, we provide stimulus materials that can support a wide range of model-based cognitive investigations. The resulting MDS-derived dimensions presented in the next section will allow researchers to test theoretical predictions across domains that differ in the relative contributions of perceptual similarity and conceptual reasoning.

## Study 1: Similarity ratings and MDS representation

## Method

### Design and procedure

After providing informed consent, participants were informed that the study aimed to investigate how individuals judge the similarity among 80 items within a given domain (food, countries, or mammals). Before the main task, participants were shown all 80 stimuli from their assigned domain to familiarize themselves with the set. In each trial of the similarity-rating task, two randomly selected stimuli were presented on the left and right of the center of the screen. In the food and mammal domains, the name of each item was shown below its corresponding image. In the country domain, only the countries’ names were presented. The sequence of stimulus pairs and the side (left or right) on which each stimulus appeared were randomized in each trial. Participants were asked to rate the similarity of each pair on a seven-point scale (1 = not similar, 7 = very similar). The similarity instructions were kept intentionally vague to avoid biasing participants toward a specific subset of features (e.g., only perceptual or semantic features). While naturalistic stimuli theoretically possess an almost infinite number of features, people rely on a finite, manageable subset of properties to form their mental representations in any given context (Medin & Ortony, 1989; Hebart et al., 2020). By providing naturalistic stimuli without overly specific instructions, we wanted to allow the set of dimensions most psychologically salient in these domains to emerge naturally.

To reduce participant burden, a balanced incomplete block design was employed: each participant rated a subset of m=158 pairs (5% of the M=3160 possible pairs). Using this procedure, each pair was rated by an average of 30.2 (SD=3.3) participants in the food domain, 25.7 (SD=3.4) in the country domain, and 33.4 (SD=5.9) in the mammal domain.

At the end of the experiment, participants rated their general knowledge of the respective domain on a seven-point scale (1 = very low, 7 = very high). They were also asked, in an open-ended format, to describe the features or cues they used when making their similarity judgments. In addition, participants provided demographic information and indicated whether their data should be included in the analysis or excluded, following the procedure described by Aust, Diedenhofen, Ullrich, and Musch (2013). They were also asked whether they had used any aids (e.g., note-taking, Internet searches), whether they had experienced any technical difficulties, and whether they had completed the study without interruption.

Additionally, three attention-check trials were embedded within the task. In each attention-check trial, a comparison pair consisted of two identical stimuli, and participants were instructed to press a designated button when they detected the identity of the two stimuli.

### Participants

All participants were recruited via Prolific. Eligibility criteria required participants to (1) be fluent in English, (2) be at least 18 years old, and (3) have an approval rating above 95%. The study took approximately 15 min to complete, and the participants were compensated £ 2.50.

We initially recruited N=757 participants for the food domain, N=787 for the country domain, and N=734 for the mammal domain. Participants were excluded if they met any of the following criteria: (1) failed at least one of the three attention checks (excluded: n=108/135/52 for food/country/mammal), (2) indicated that their data should not be used for analysis (n=4/10/2), (3) reported cheating (e.g., taking notes, using online resources, or consulting others; n=2/22/1), (4) had a correlation below r=.25 between their individual similarity ratings and the group average, following Nosofsky et al. (2018c) (n=35/98/7), or (5) responded in under 400 ms on more than 10% of trials (n=1/2/1). After exclusions, the final sample comprised N=607 participants in the food domain (age: M=39.8, SD=12.8, range = 18–76; 43.5% female), N=520 in the country domain (age: M=38.6, SD=13.3, range = 18–79; 48.1% female), and N=671 in the mammal domain (age: M=40.9, SD=13.3, range = 18–77; 44.0% female).

**Fig. 2:**
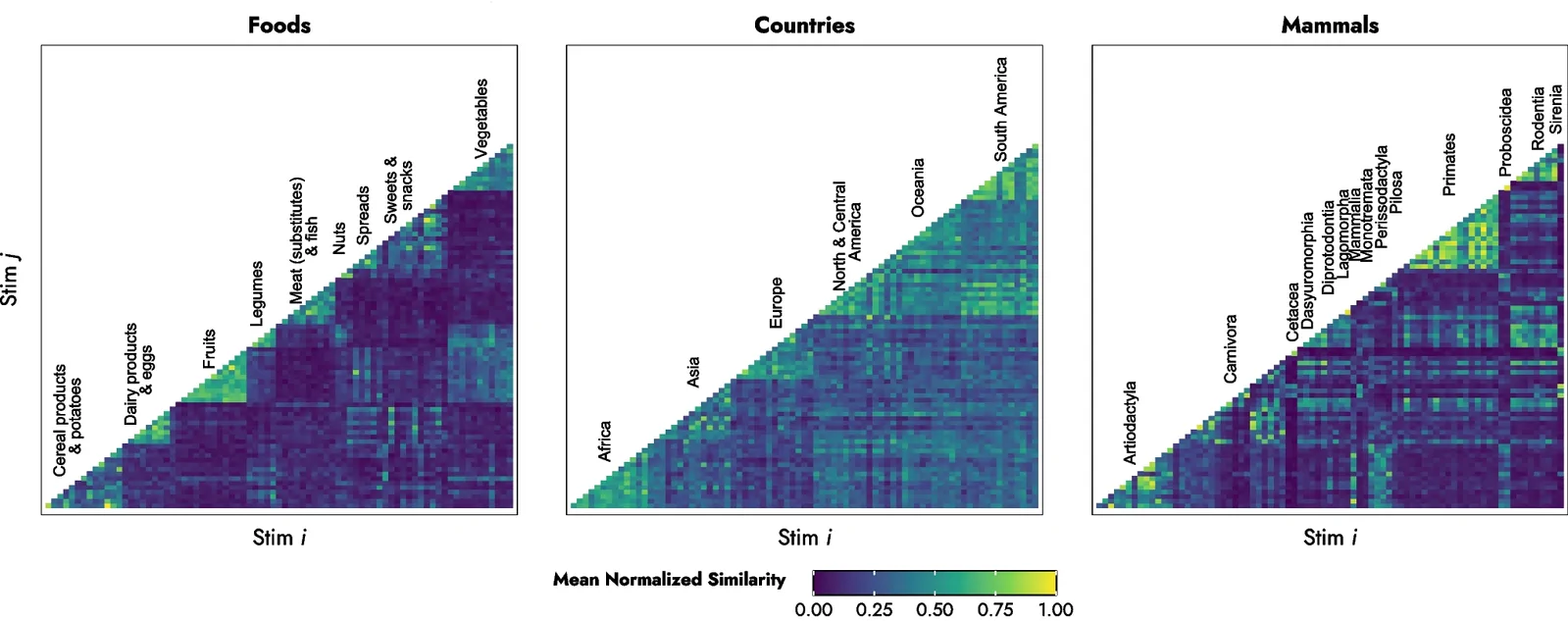
Average pairwise similarities between all 80 stimuli in each domain, grouped by ad-hoc stimulus categories

**Table 1:** Five most similar and dissimilar stimulus pairs (on average) in each domain

Stimulus *i*	Stimulus *j*	*M*
**(a) Food**		
Bell pepper	Whipped cream	.00
Salmon	Chocolate	.00
Watermelon	Pork	.01
Fried egg	Gummy bears	.01
Chicken	Chocolate cake	.01
Gouda cheese	Mozzarella	.86
Bread roll	Bread	.86
Chocolate	Chocolate bar	.95
Orange	Tangerine	.95
Bread	Toast	.99
**(b) Countries**		
Iran	Jamaica	.04
Nigeria	Ireland	.04
India	Netherlands	.05
China	Ireland	.06
China	Cook Islands	.06
Peru	Chile	.81
Germany	France	.81
Iran	Syria	.82
Russia	Ukraine	.83
United States	Canada	.86
**(c) Mammals**		
Red panda	B. dolphin	.00
Dromedary	H. whale	.00
Lion	H. whale	.00
B. dolphin	Brush-tailed possum	.00
B. dolphin	Stump-tailed macaque	.00
Tufted capu	Proboscis monkey	.95
Mountain hare	European rabbit	.96
African bush elephant	Asiatic elephant	.96
Stump-tailed macaque	Chimpanzee	.96
Stump-tailed macaque	Japanese macaque	.99

At the trial level, we excluded (1) trials in which participants pressed the button reserved for attention checks (k=49/43/119), (2) trials with response times below 400 ms (k=79/56/47), and (3) trials exceeding 30 seconds (k=385/958/315). After these exclusions, the final dataset included $K = 95{,}393$ trials in the food domain, $K = 81{,}103$ in the country domain, and $K = 105{,}695$ in the mammal domain.^3^

## Results

All analyses were conducted using the statistical software R (Version 4.4, R Core Team, 2023). The analysis scripts and data needed to reproduce the results are available on OSF. For the subsequent analyses, we first averaged participants’ similarity ratings for each stimulus pair. These average ratings were then normalized to a scale of 0–1.

### Descriptive results of similarity ratings

On average, normalized similarity ratings (scaled from 0 to 1) were higher in the country domain (*M* = .33, *SD* = .15, range = .04–.86) compared to the food (*M* = .17, *SD* = .16, range = .00–.99) and mammal (*M* = .21, *SD* = .19, range = .00–.99) domains. Figure 2 displays the average pairwise similarities between all 80 stimuli within each domain, along with ad hoc categories (e.g., food groups, continental regions, mammal orders)^4^. In addition, Table 1 presents the five most similar and most dissimilar stimulus pairs per domain, based on participants’ average similarity ratings.

**Fig. 3:**
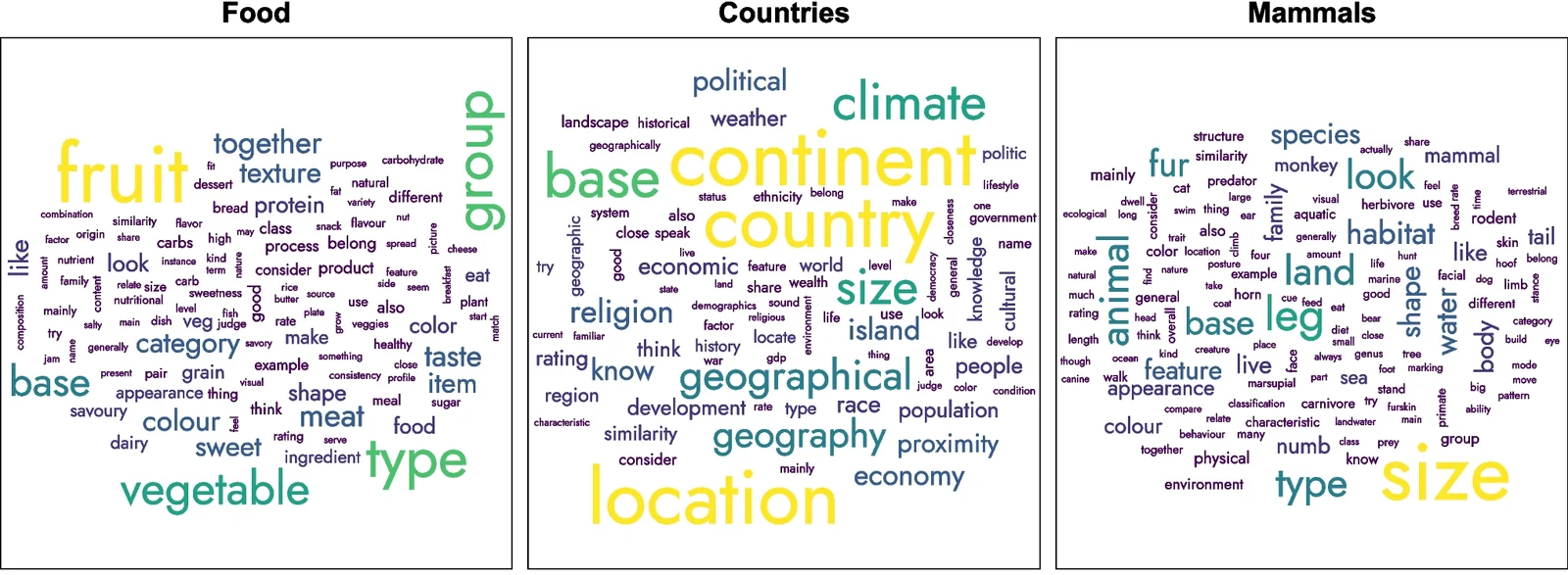
Word cloud visualization of features participants reported using to judge similarity across domains. *Note.* Word size reflects relative frequency of mention

Based on participants’ open-ended responses, Fig. 3 provides a summary of the features they reported using to make similarity judgments. Responses were preprocessed by converting all text to lowercase, removing punctuation, extra whitespace, and filler words (e.g., "the", "whether", etc.), and then lemmatized (e.g., “culture” and “cultural” were mapped to “culture”). Only features mentioned by at least five participants are shown.

### Reliability

Following previous work (Verheyen, Ameel, & Storms, 2007; Verheyen & Storms, 2021), we estimated the reliability of the aggregated similarity data in each domain as the mean split-half correlation corrected with the Spearman–Brown formula, across 1000 random splits. Reliability estimates were high in all domains: food (M=.94, SD<.01), countries (M=.87, SD<.01), and mammals (M=.97, SD<.01), indicating stable similarity structures suitable for subsequent multidimensional scaling analyses. In addition, a permutation test implemented in the smacof package (Mair, Groenen, & de Leeuw, 2022) confirmed that observed stress values were significantly lower than expected under random dissimilarities (all *p* <.001), indicating substantial structure in the similarity data.

### Individual differences in similarity judgments

Aggregating similarity judgments across participants before multidimensional scaling analysis is common practice in the literature (e.g., Nosofsky et al., 2018c; Shepard, 1962b; Shin et al., 1992). Such aggregation is also often necessary, particularly in similarity rating tasks that do not rely on exhaustive pairwise comparisons (e.g., the Spatial Arrangement Method; e.g., Goldstone, 1994; Hout et al., 2013; Verheyen & Storms, 2021), and becomes practically unavoidable in large stimulus sets such as the present one, where the number of possible item pairs makes it infeasible to obtain complete individual-level similarity matrices for each participant.

To assess the extent of individual differences in similarity structure, we computed two complementary indices. First, for each participant, we calculated the correlation between their similarity ratings and the corresponding group-average similarity ratings (leave-one-out). This measure provides an estimate of how closely individual judgments align with the aggregate similarity structure. Second, we estimated the intraclass correlation coefficient (ICC) based on an intercept-only multilevel model with random intercepts for participants and stimulus pairs, thereby quantifying the proportion of variance attributable to shared versus idiosyncratic components of similarity judgments. The stimulus-pairs-level ICC reflects the extent to which specific stimulus pairs are judged similarly across participants, and thus provides an index of shared representational structure (since similar dimensions should lead to a similar similarity rating). In contrast, the person-level ICC captures stable participant-specific response tendencies (e.g., generally using higher or lower similarity ratings).

Across domains, correlations between individual and group-level similarity structures indicated substantial, but not perfect, agreement. Mean correlations were highest in the mammals domain (M=.77, SD=.10), followed by the food domain (M=.69, SD=.14), and lowest in the countries domain (M=.54, SD=.13)^5^. These results suggest that, while a robust common similarity structure exists (as also indicated by the previous reliability analysis), individuals differ in how strongly their judgments align with the aggregate representation.

A similar pattern emerges from the ICC analyses. Stimulus-pairs ICCs were moderate to high (.35, .22, and .49 for food, countries, and mammals, respectively), whereas person-level ICCs were consistently lower (.20, .19, and .16)^6^.

Taken together with the reliability analyses reported above, these results suggest that the MDS solutions derived from aggregated data capture a robust common similarity structure. At the same time, there remain meaningful differences between aggregate and individual-level similarity patterns. These differences should be interpreted with some caution, as each participant contributed only a subset of all possible pairwise judgments (approximately 5%), which may introduce additional variability in individual-level estimates. Nevertheless, the results indicate that individual differences are more pronounced in the country domain compared to the food and mammal domains, where similarity judgments show stronger alignment across participants.

### Multidimensional scaling

We employed standard non-metric multidimensional scaling (MDS) as a well-established tool to derive similarity-based representations of the real-world stimuli that can serve as cues or attributes in formal models of cognition.^7^

For the multidimensional scaling analysis, we subtracted the normalized similarities from one to obtain dissimilarities, which is the required dependent variable for most MDS algorithms. To perform an MDS analysis, one has to specify the number of dimensions the final MDS configuration should have in advance, and several methodological approaches have been proposed to determine the appropriate dimensionality (e.g., Borg & Groenen, 2005; Gronau & Lee, 2020; Lee, 2001; Verheyen et al., 2007). Based on the results of previous studies (Izydorczyk & Bröder, 2024; Richie, White, Bhatia, & Hout, 2020; Steyvers, 2006), we decided to use a leave-one-out cross-validation (loo-cv) procedure (Hastie, Tibshirani, & Friedman, 2009) to determine the optimal dimensionality and to avoid overfitting. Specifically, for a given number of dimensions *D*, we iteratively removed one unique pairwise dissimilarity from the matrix, computed a non-metric MDS solution of dimensionality *D* based on the remaining entries using the smacof package, and then predicted the left-out dissimilarity based on the resulting configuration. This process was repeated for all unique stimulus pairs (i.e., the lower-triangular entries of the matrix). Prediction accuracy was quantified using the root-mean-square error (RMSE) across all leave-one-out iterations. The procedure was repeated for dimensionalities ranging from 1 to 20, and the optimal number of dimensions was defined as the one that yielded the lowest RMSE. According to this leave-one-out cross-validation procedure, the optimal number of dimensions was *D* = 14 for the food domain, *D* = 10 for the country domain, and *D* = 10 for the mammal domain. Full results of the loo-cv analysis are reported in Table 5 in the Appendix.

**Fig. 4:**
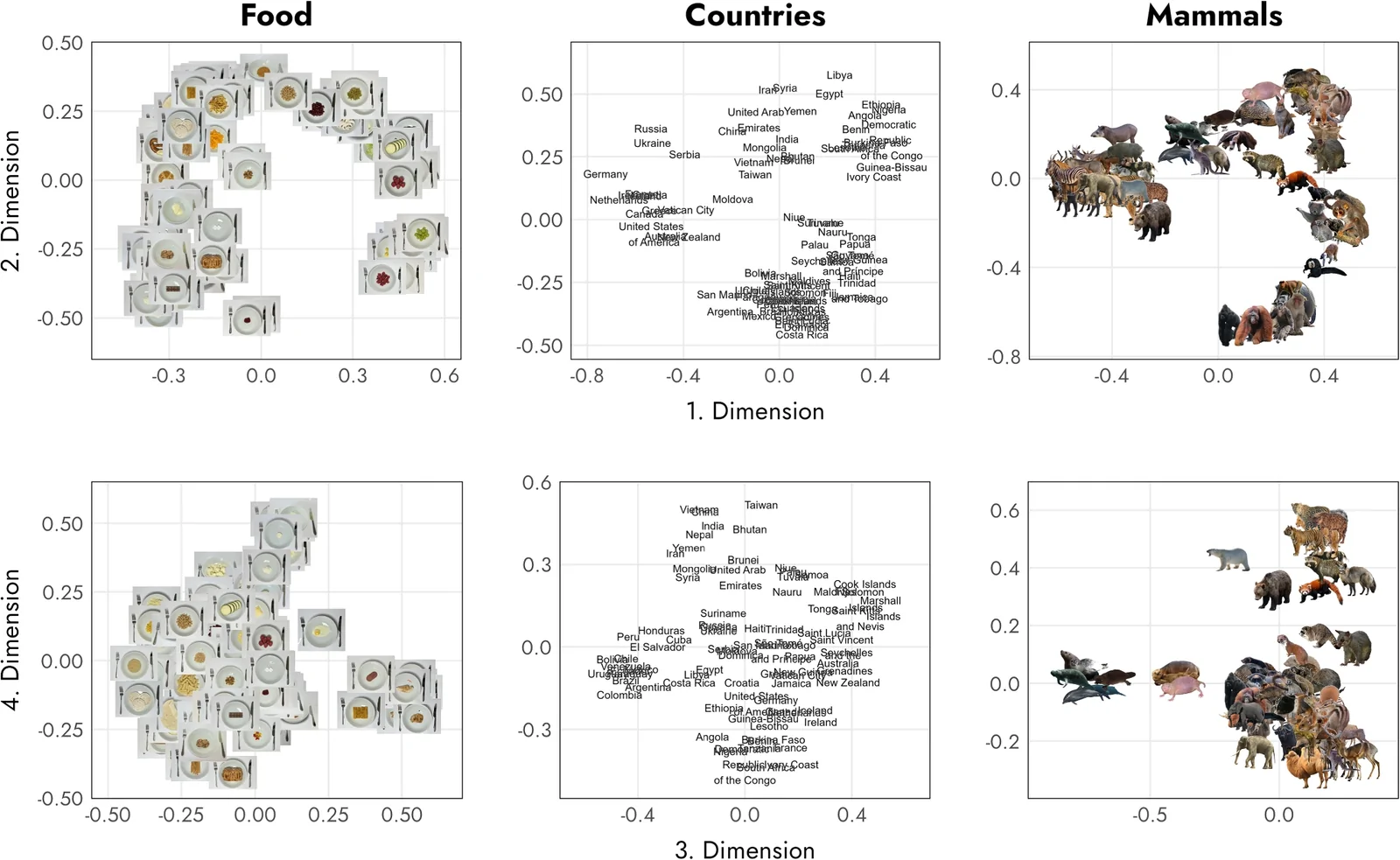
First four dimensions of the final MDS configuration for each domain

We then computed the final MDS configuration for each domain using non-metric multidimensional scaling with principal axis transformation, as implemented in the smacof package (Mair et al., 2022), which assumes a Euclidean distance function, a monotonically increasing (non-metric) transformation function, and the stress-1 loss function. Each solution used the optimal number of dimensions identified via leave-one-out cross-validation^8^. The stress-1 values of the final solutions were 0.04 (food), 0.07 (countries), and 0.05 (mammals). The correlations between the observed empirical dissimilarities and those predicted by the corresponding MDS configurations were high across all domains: *r*(3158) = .97 for the food domain, *r*(3158) = .93 for the country domain, and *r*(3158) = .97 for the mammal domain (all *p*s < .001), indicating that the MDS configurations provided a very good reconstruction of the similarity space in each domain. Figure 4 illustrates the spatial arrangement of all 80 stimuli in the first four dimensions^9^ of each domain’s MDS solution. Although interpreting the MDS dimensions was not the primary aim of our analysis, preliminary inspection and analyses reported in the Supplementary Materials suggest that the first few dimensions captured broad thematic or categorical distinctions^10^. For example, in the mammal domain, Dimension 3 appeared to separate land-dwelling from sea-dwelling mammals, whereas in the country domain, Dimension 1 roughly reflected differences related to national development (e.g., life expectancy, well-being, human development index, etc.).

## Study 2: Proof-of-relevance

The extraction of stimulus dimensions is a first step toward deriving attributes or cues that may be relevant as input for modeling other cognitive processes, such as categorization, estimation, or judgment. However, there is no a priori guarantee that the dimensions that govern similarity judgments also form the basis of other judgment processes or categorization. Hence, a second step of validation is to show that the extracted features also predict other judgments and therefore can be used as a basis for modeling these processes (see Izydorczyk & Bröder, 2024; Nosofsky, Sanders, & McDaniel, 2018b; Smits et al., 2002). Therefore, we collected quantitative judgments for all stimuli within each domain, asking participants to estimate domain-relevant criterion values. Below, we first provide a brief description of the experimental procedure and then summarize the results concerning (a) participants’ estimation performance and (b) the extent to which the MDS-derived dimensions capture both the objective criterion structure of the environment and participants’ estimates at the individual and aggregate levels. A full description of the training procedure, stimuli, and estimation tasks can be found in the supplementary materials online and the companion paper with more in-depth analysis, testing various cognitive models of estimation.^11^

### Method

Participants completed a judgment experiment consisting of a training phase followed by a testing phase (e.g., Hoffmann et al., 2016; Juslin et al., 2003). After providing informed consent, participants were trained to estimate a domain-specific criterion: grams of carbohydrates per 100 g for food items (*N* = 46), average life expectancy for countries (*N* = 48), or days until female maturity for mammals (*N* = 48). The criteria were chosen to differ in familiarity, prior knowledge, and numerical scale, enabling comparisons across diverse estimation environments. During training, participants repeatedly estimated criterion values for a subset of k=12 exemplars and received immediate feedback after each response. Training lasted at least five blocks and continued until participants achieved an accuracy criterion (at least ten of 12 estimates within ±10% of the true value) or completed a maximum of ten blocks. In the subsequent testing phase, participants estimated the criterion values for all 80 stimuli (12 trained exemplars and 68 novel items) presented in randomized order without feedback.

### Results

All analyses reported below focus exclusively on the *k* = 68 new items presented during the testing phase, that is, excluding the *k* = 12 exemplars participants encountered during training. Analyses were conducted in R (Version 4.4, R Core Team, 2023). Data and analysis scripts required to reproduce all results are available on OSF.

#### Estimation performance

To assess estimation accuracy, we examined how closely the average subjective estimates for the test items matched the true criterion values in each domain. Across all domains, the mean estimates showed strong positive correlations with the true criterion values (life expectancy of countries: r(66)=.84, p<.001; carbohydrate content for food items: r(66)=.70, p<.001; days until female maturity of mammals: r(66)=.72, p<.001). Overall, these results indicate that, on average, participants learned meaningful relationships between the stimuli and the criterion values during training and generalized this knowledge to previously unseen items.

#### Validity of MDS dimensions

To evaluate the validity of the MDS dimensions, we used a regression-based approach inspired by Brunswik’s lens model (Brunswik, 1952). Specifically, we conducted regression analyses using all available MDS dimensions as predictors (i.e., *D* = 14 for food, *D* = 10 for countries and mammals). Two types of models were estimated: (a) an environmental model predicting the true criterion values and (b) a person-level model predicting participants’ estimates.

##### Environmental-level analysis

When predicting the true criterion values, the MDS dimensions accounted for a substantial proportion of variance: adjusted $R^2$^12^ = .73 for food, $R^2$ = .73 for countries, and $R^2$ = .66 for mammals. Thus, dimensions obtained from pairwise similarity ratings captured meaningful structural information about the respective domains.

##### Person-level analysis

Next, we assessed how well these MDS dimensions accounted for participants’ subjective estimates. At the aggregate level, the dimensions explained a large proportion of variance in the average estimates for new items, with adjusted $R^2 =.87$ for food, $R^2 =.88$ for countries, and $R^2 =.76$ for mammals.

**Fig. 5:**
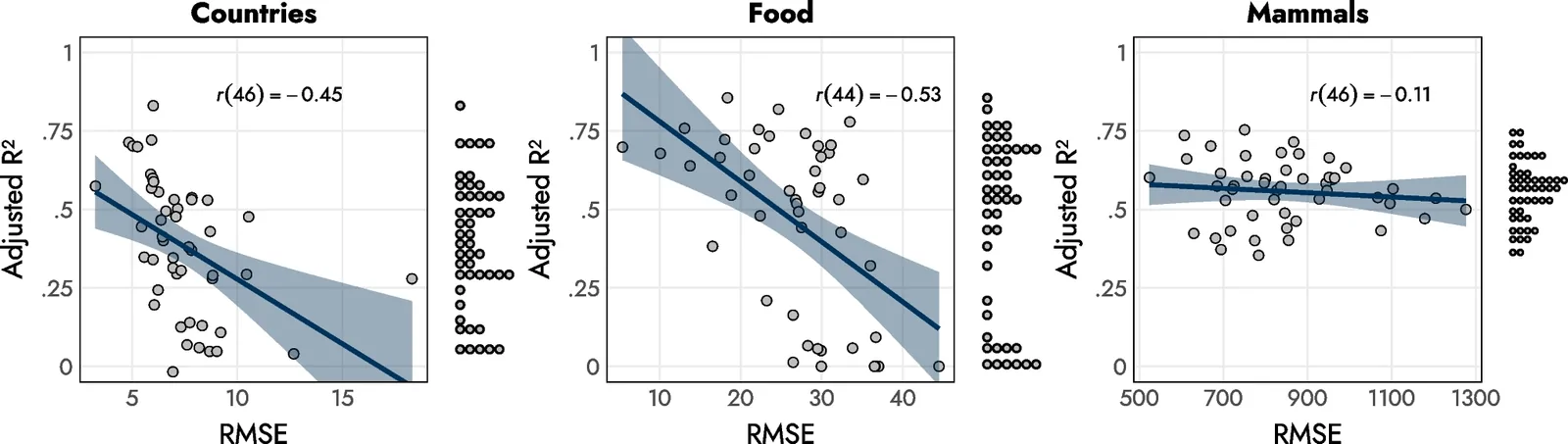
Distribution of adjusted $R^2$ values of participants in each domain. *Note.* Relationship between estimation accuracy (root mean squared error; RMSE) and explained variance (adjusted $R^2$) from participant-level regression models predicting individual estimates from MDS dimensions across the three domains. Each *point* represents one participant. The main panels display the association between RMSE and adjusted $R^2$, whereas the marginal plots show the distribution of adjusted $R^2$ values within each domain

At the individual level, separate linear models were fitted for each participant using the MDS dimensions as additive predictors of their estimates. Across participants, the dimensions explained a substantial amount of variance in individual estimates: the average adjusted $R^2$ was *M* = .46 (*SD* = . 29, range = – .07–.86]) for food; *M* = .38 (*SD* = .21, range = – .02–.83) for countries; and *M* = .56 (*SD* = .10, range = .35–.75) for mammals. As shown in Fig. 5, variability in explained variance across participants (see marginal plots of Fig. 5)was partially due to the difference in estimation accuracy of participants. In the countries (r(46)=-.45, p=.001) and food (r(44)=-.44, p<.001) domains, participants with lower RMSE between true and estimated values showed higher adjusted $R^2$ values. No such relationship was observed for mammals (r(44)=-.11, p=.44).

### Discussion

These findings suggest that the dimensions derived from pairwise similarity judgments captured structurally relevant aspects of the respective domains and, importantly, also reflect psychologically meaningful cues that people rely on when forming judgments. Thus, the MDS dimensions appear to approximate both environmental regularities and internal representations that generalize across cognitive tasks.

At the individual level, variability in explained variance indicates substantial differences in how strongly participants’ estimates aligned with the structure captured by the MDS dimensions. Part of this variability was associated with estimation accuracy: participants who produced more accurate estimates tended to show higher adjusted $R^2$ values, suggesting that successful learning during training increased reliance on domain structure represented by the dimensions. One interpretation is that participants who engaged less with the learning phase or relied more on guessing during testing produced responses that could not be well described by the structural information encoded in the MDS space. Interestingly, this relationship between estimation accuracy and explained variance was absent in the mammals domain. One possible explanation is that this domain was particularly engaging or intuitive for participants, leading to more consistent task engagement across individuals and thereby reducing variability in the alignment between judgments and dimensional structure. However, the lower explained variance for some participants may also reflect that these participants relied on cues for their estimates which are not captured by the MDS dimensions, either because of the difference of the task (similarity ratings vs. criterion estimation, Thibaut, Dupont, & Anselme, 2002) or because of individual differences in representations used to make similarity ratings (e.g., Ashby, Maddox, & Lee, 1994; Medin, Lynch, Coley, & Atran, 1997; O’hare, 1976). Finally, some participants may have relied on cognitive processes not well captured by a linear additive combination of MDS dimensions. For instance, participants may have relied on exemplar-based mechanisms (Hoffmann et al., 2016; Juslin & Persson, 2002; Juslin et al., 2003) or mapping processes (von Helversen & Rieskamp, 2008, 2009), which can produce estimates that deviate from linear cue-integration models.

## Summary and general discussion

Theorizing in Cognitive Psychology has reached a high level of formalization with computational theories or models for many cognitive processes, such as categorization, memory, and judgment (Busemeyer & Diederich, 2010; Farrell & Lewandowsky, 2018; Lamberts, 2005). This theoretical sophistication often comes at the price of relying on highly simplified stimulus materials, as formal models typically require well-defined and explicitly specified stimulus attributes. Hence, while theories of cognition claim generality, their empirical testing is often confined to simplified stimulus environments.

Based on previous work (e.g., Nosofsky et al., 2018b; Nosofsky et al., 2018c; Smits et al., 2002; Storms et al., 2001), the current work addresses this gap by providing a well-curated set of naturalistic stimuli from three domains, together with similarity data and derived embeddings. By collecting over 280,000 similarity ratings, using non-metric multidimensional scaling, we derived similarity-based representations that can serve as input features for computational models. Importantly, the contribution is not methodological in nature, but lies in the construction and provision of a well-curated resource consisting of naturalistic stimuli, similarity judgments, and derived representational spaces.

The present database thus provides researchers with the necessary attributes to test cognitive models using more realistic and ecologically valid stimuli. The food and the country domain form knowledge domains of significant societal importance. Given that nutrition and food literacy are critical factors in public health (Kilb, Giese, & Mata 2023; Silva, Araújo, Lopes, & Ray, 2023), precise cognitive modeling of knowledge acquisition in this domain may facilitate the design of effective interventions. Similarly, geopolitical knowledge is of growing relevance in an increasingly complex multilateral world. Although mammals may be less prevalent in everyday contexts, this biological domain was included to connect with early categorization research on natural stimuli (Rosch, Mervis, Gray, Johnson, & Boyes-Braem, 1976) and because it might be more engaging stimulus material for participants.

Although we consider the present collection of stimuli and their dimensions a useful starting point for transferring cognitive research from the lab to the real world, there are obvious limitations with our approach.

First of all, our sample was likely WEIRD in the sense of Henrich, Heine, and Norenzayan (2010). Food items and the cognitive representations of countries in particular may reflect a specific Western profile that could be quite different in, for example, predominantly African or Islamic countries. For example, pork may be virtually absent in Islamic countries, and potatoes might be less popular in some African countries. This is likely to result in different cognitive representations of our food items. The distortions may be even greater for the country representations. It may also extend to the mammals category, for which the place you live in probably determines how differentiated you perceive certain animals (e.g., several types of antelopes in Africa).

This preliminary focus on rather Western cultural stimuli (concerning food) and participant samples was deliberate in the current case: The alternative would have been to choose less distinctive foods that are common in most cultural contexts and to elicit similarity judgments from a broad variation of cultures. However, we decided against this option because an "average" cognitive space across very different representations might have resulted in less meaningful dimensions than a space generated by a relatively homogeneous group of participants from one cultural background. Our approach was therefore to begin with a relatively precise and culturally coherent representation that is restricted to one cultural background rather than a broad average that may appear to be more broadly applicable at first sight, but is eventually deprived of meaningful dimensions.

A second caveat, of course, is that the demonstration of the predictive power of extracted dimensions for the estimation tasks in our second study certainly does not guarantee that the dimensions are relevant in *all* cognitive tasks. Rather, some dimensions may be more relevant than others in different tasks, or specific tasks may not be related to the dimensions at all. Although we consider this unlikely, we would recommend brief pilot studies for new tasks to get a preliminary impression of whether the dimensions can be used for modeling in principle. If the dimensions do not predict behavior in any way, attempts to model behavior using these dimensions might be futile.

A third limitation of the present study concerns participants’ familiarity with the stimuli. Participants were not asked to indicate whether they were familiar with individual items explicitly. This issue is particularly relevant for the country domain, where similarity ratings arguably primarily reflect semantic knowledge about countries rather than perceptual information. The comparatively higher exclusion rate observed in the country domain may partly reflect this difference, as participants with limited geographic knowledge may have been less able or less motivated to meaningfully engage with the task. Future studies using similar stimulus sets should therefore include post-task familiarity ratings or recognition checks to assess participants’ knowledge of individual stimuli and to distinguish informed judgments from potential guessing behavior. Incorporating such measures would allow researchers to more directly examine how familiarity moderates similarity judgments and could further improve data quality and interpretability.

Another limitation is the nature of the recovered similarity space. In the judgment task of Study 1, the term similarity was deliberately left unspecific to encompass the full range of potential dimensions, whether semantic or perceptual. We view this integration as a core strength of the approach. For naturalistic stimuli like food or mammals, sensory features and semantic knowledge are intrinsically coupled, this is, a picture of an animal automatically triggers its conceptual category, just as a category label evokes visual mental images. Historically, research has distinguished between continuous spatial models for perceptual stimuli and discrete featural models for conceptual ones (Pruzansky, Tversky, & Carroll, 1982; Tversky & Hutchinson, 1986). Although we utilized MDS to recover continuous dimensions, we acknowledge that different representational geometries might capture specific aspects of the data better. For instance, hybrid models that explicitly combine continuous dimensions with discrete, overlapping features have been shown to provide a more flexible and psychologically plausible account for stimuli that span perceptual and conceptual domains (e.g., Navarro & Lee, 2019). Furthermore, while naturalistic stimuli theoretically possess an almost infinite number of characteristics, people typically rely on a finite, manageable subset of properties to form their mental representations in a given context (Hebart et al., 2020; Medin & Ortony, 1989). By providing naturalistic stimuli without restrictive guidance, we allow the most psychologically salient features to emerge, which, depending on the domain and context, can be more semantic or perceptual. This approach aims to capture the features that are most psychologically salient across a broad range of tasks. How these different features are weighted in cognitive tasks using the stimuli may depend on the modeled task, and this will likely be reflected in the importance parameters of the respective cognitive models. Also, this mixture of feature types is consistent with modern computational advances, where multimodal neural networks demonstrate that aligning visual and linguistic signals into a shared embedding space yields more robust and human-like representations than unimodal models (Battleday et al., 2020). Nevertheless, we acknowledge that for certain specific research questions, it might be worthwhile to work with more precisely defined concepts, such as "perceptual similarity" for mammals or "cultural similarity" for countries. A targeted focus would likely change the representation reconstructed by MDS, potentially yielding fewer but more specialized dimensions. In such cases, we encourage researchers to use our stimuli to elicit their own MDS representations from more targeted similarity judgments.

A final limitation concerns the assessment of individual-level similarity structures. Given the very large number of possible stimulus pairs, each participant provided only a small subset of similarity ratings (approximately 5% of all pairs), such that the reported MDS solutions are necessarily based on aggregated data. While this aggregation approach is standard in the literature, it may obscure meaningful individual differences in underlying representations (Ashby et al., 1994). We sought to quantify these differences through the analyses of individual-level agreement and reliability reported above, which indicated the presence of a substantial shared structure across participants. Nevertheless, these results also suggest that individual representations may deviate from the aggregate similarity space for a subset of participants. Researchers using the present data should therefore be mindful that the provided MDS solutions reflect group-level structure and may not equally well capture all individual representations. One potential avenue for future work would be to obtain additional similarity judgments for a targeted subset of stimulus pairs at the individual level and use these data to adapt the aggregate MDS space, for example by applying individual-specific weighting schemes or augmenting the representation with additional dimensions.

Despite these limitations, we hope that researchers will find our freely shared stimuli, similarity data, and the extracted dimensions useful as a starting point for modeling cognitive processes. We encourage all to add further validation attempts or norming data of these stimuli to the database by mailing the respective datasets to one of the current authors.

## Supplementary Information

Below is the link to the electronic supplementary material.

Supplementary file 1 (pdf 161 KB)

## Data Availability

The materials and data are freely available on the GitHub and OSF repositories of this project.

## Appendix A: Item lists

**Table 2:** List of all 80 food items

Item	Item	Item	Item
Spaghetti	Cauliflower	Gouda cheese	Chocolate
Rice	Mushrooms	Butter	Vanilla pudding
Rigatoni	Peas	Feta	Chips
Bread roll	Lentils	Mozzarella	Pretzel sticks
Oat flakes	Beans	Cream cheese	Apple turnover
Couscous	Banana	Salami	Chocolate cake
Bread	Apple	Dried ham	Cheesecake
Croissant	Watermelon	Vienna sausages	Cookies
White toast	Kidney beans	Tuna (canned)	Peanuts
Bulgur	Orange	Shrimps	Walnuts
Wrap	Mandarin	Salmon	Whipped cream
Potatoes	Mango	Pork	Chickpeas
Cucumber	Pineapple	Chicken	Radishes
Bell pepper	Strawberry	Nutella	Fish sticks
Carrots	Raspberry	Cherry jam	Vegetarian salami
Corn	Pear	Raspberry jam	French fries
Fried egg	Apricot	Honey	Chocolate bar
Broccoli	Grapes	Peanut butter	Eggplant
Tomatoes	Yogurt	Gummy bears	Rice cake
Iceberg lettuce	Curd cheese	Ice cream	Dextrose

**Table 3:** List of all 80 countries

Item	Item	Item	Item
China	Angola	United Arab Emirates	Suriname
India	Peru	Libya	**Brunei**
United States of America	Yemen	El Salvador	Iceland
Nigeria	Ivory Coast	Serbia	Maldives
Brazil	Nepal	Paraguay	Sao Tome & Principe
Russia	Venezuela	Ireland	Samoa
Mexico	Australia	New Zealand	Saint Lucia
Ethiopia	Taiwan	Costa Rica	Saint Vincent & the Grenadines
Egypt	Syria	Croatia	Seychelles
Vietnam	Burkina Faso	Mongolia	Tonga
Democratic Republic of the Congo	Chile	Uruguay	Dominica
Germany	Netherlands	Jamaica	Saint Kitts & Nevis
Iran	Ecuador	Moldova	Marshall Islands
France	Benin	Lesotho	San Marino
South Africa	Bolivia	Guinea-Bissau	Palau
Tanzania	Papua New Guinea	Trinidad & Tobago	Cook Islands
Colombia	Haiti	Fiji	Nauru
Argentina	Cuba	Bhutan	Tuvalu
Canada	Greece	Guyana	Niue
Ukraine	Honduras	Solomon Islands	Vatican City

**Table 4:** List of all 80 mammals

Item	Item	Item	Item
Aoudad	Raccoon dog	Western gray kangaroo	Japanese macaque
Brazilian tapir	Bat-eared fox	Quokka	Gorilla
American bison	Cheetah	Sugar glider	Chimpanzee
Giant anteater	Lion	Brush-tailed possum	Orangutan
Ibex	Leopard	Koala	Brown lemur
Thomson’s gazelle	Tiger	Mountain hare	Black lemur
Llama	Dwarf mongoose	European rabbit	Ring-tailed lemur
Kirk’s dik-dik	Meerkat	Duck-billed platypus	Slender loris
Red panda	Spotted hyena	Short-beaked echidna	white-faced saki
African buffalo	Sea otter	Horse	African bush elephant
Bactrian camel	Least weasel	Quagga	Naked mole-rat
Dromedary	Steller sea lion	White rhinoceros	Guinea pig
Mule deer	Harbor seal	Central American spider monkey	Degu
Caribou and reindeer	Common raccoon	White-tufted-ear marmoset	Lesser Egyptian jerboa
Giraffe	Brown bear	Midas tamarin	Long-tailed field mouse
Hippopotamus	Polar bear	Tufted capu	Nutria
Wart hog	Humpback whale	De Brazza’s monkey	Eurasian red squirrel
Wild boar	Bottlenosed dolphin	Guereza	Siberian chipmunk
Gray wolf	Australian tiger cat	Stump-tailed macaque	Dugong
African wild dog	Tasmanian devil	Proboscis monkey	Asiatic elephant

## Appendix B: Results of leave-one-out cross-validation

**Table 5:** Results of leave-one-out cross-validation

# Dim	$R^2$	RMSE
**(a) Food**		
1	.231	0.481
2	.552	0.327
3	.686	0.262
4	.741	0.228
5	.781	0.208
6	.794	0.196
7	.805	0.185
8	.807	0.180
9	.799	0.172
10	.809	0.168
11	.795	0.163
12	.803	0.161
13	.813	0.159
**14**	**.823**	**0.159**
15	.819	0.160
16	.799	0.164
17	.788	0.166
18	.773	0.169
19	.752	0.174
20	.730	0.176
**(b) Countries**		
1	.399	0.474
2	.617	0.392
3	.661	0.362
4	.709	0.342
5	.716	0.335
6	.700	0.330
7	.712	0.327
8	.703	0.325
9	.684	0.323
**10**	**.677**	**0.322**
11	.665	0.323
12	.655	0.323
13	.654	0.323
14	.628	0.325
15	.607	0.326
16	.630	0.326
17	.570	0.328
18	.619	0.328
19	.591	0.330
20	.560	0.332
**(c) Mammals**		
1	.367	0.457
2	.638	0.330
3	.746	0.269
4	.797	0.242
5	.847	0.220
6	.861	0.208
7	.876	0.202
8	.879	0.195
9	.887	0.192
**10**	**.884**	**0.184**
11	.894	0.186
12	.899	0.186
13	.890	0.188
14	.892	0.189
15	.888	0.193
16	.891	0.194
17	.876	0.200
18	.866	0.204
19	.865	0.206
20	.869	0.208

*Note.* Presented values are rounded to the third decimal. Lowest RMSE values are shown in bold

## Appendix C: Approximate explained variance similarity data by MDS dimensions

Because we employed ordinal (non-metric) multidimensional scaling, a decomposition of variance explained by individual dimensions, analogous to eigenvalues in principal component analysis, is not formally defined. In non-metric MDS, the solution is optimized with respect to a monotonic transformation of the dissimilarities, and model fit is evaluated using stress rather than variance accounted for.

To obtain an interpretable approximation of the relative contribution of each dimension, we computed a distance-based $R^2$ measure reflecting how well the MDS configuration reproduces the original dissimilarities. Specifically, we first calculated the squared correlation between the original similarities and the predicted distances of the full MDS solution. We then iteratively recomputed this $R^2$ after removing one dimension at a time from the configuration. The contribution of a given dimension was then defined as the decrease in $R^2$ relative to the full solution: $\Delta R_i^2=R_{\text{ full } }^2-R_{(-i)}^2$, where $R_{(-i)}^2$ denotes the fit of the configuration with dimension *i* removed. This leave-one-dimension-out procedure provides a pragmatic, albeit approximate, index of the relative importance of each dimension in preserving the dissimilarity structure. We emphasize that this measure does not constitute a formal variance decomposition but serves as a descriptive tool to aid the interpretation of high-dimensional MDS solutions.

The resulting dimension-wise contributions were computed separately for each stimulus domain (food, countries, mammals) and summarized in Table 6.

**Table 6:** Approximate % of explained variance of the average similarity data by each MDS dimension

# Dim	Food	Countries	Mammals
1	.37	.40	.41
2	.22	.22	.30
3	.20	.14	.24
4	.11	.11	.10
5	.07	.05	.07
6	.04	.05	.03
7	.04	.05	.03
8	.03	.03	.02
9	.02	.02	.03
10	.02	.02	.01
11	.01		
12	.02		
13	.01		
14	.01		

## Footnotes

1 Throughout the manuscript, we use the terms features, dimensions, attributes, and cues interchangeably to refer to latent properties that characterize objects within a psychological representation space. The terminology varies across research traditions in cognitive psychology: multidimensional scaling research typically refers to "dimensions" (e.g., Kruskal & Wish, 1978; Shepard, 1962a), categorization literature often uses "features" or "attributes" (e.g., Tversky, 1977; Nosofsky, 1986), judgment and decision-making research commonly adopts the term "cues" (e.g., Brunswik, 1955; Gigerenzer & Goldstein, 1996), and the machine learning literature refers to "embeddings" (Hebart, Zheng, Pereira, & Baker, 2020; Peterson, Abbott, & Griffiths, 2016). In the present context, all terms refer to inferred or defined (latent) properties of an object.

2 Many stimuli used by Izydorczyk are provided at https://osf.io/y5m7s/

3 We applied lenient exclusion criteria to retain as much data as possible while maintaining basic data quality standards. However, because these criteria were not preregistered, we conducted a sensitivity analysis to evaluate the robustness of the results. In particular, we relaxed the potentially debatable criteria based on response-time thresholds (responses faster than 400 ms or slower than 30,000 ms) and the exclusion based on low correlation with the group-average similarity pattern (Nosofsky et al., 2018c). All other criteria were retained, as they reflect clear indicators of low-quality data (e.g., failed attention checks or incomplete participation). Under these less restrictive inclusion criteria, the final samples comprised N=643, 620, and 679 participants, contributing $K = 101{,}515$, 97, 876, and 107, 312 trials in the food, country, and mammal domains, respectively. The resulting average pairwise distance matrices remained highly stable, with correlations exceeding r>.99, .98, and .99 between the original and relaxed datasets. Descriptive statistics of similarity ratings (mean, standard deviation, and range) were virtually unchanged, indicating that the reported results are robust to reasonable variations in exclusion criteria.

4 Category assignments were based on established domain-specific taxonomies whenever possible (e.g., biological classifications, geographic continents, or standardized food groupings) and were chosen to achieve a meaningful level of granularity for the present stimulus sets. For food items, category assignments were guided by the German Nutrient Database. We note that alternative, equally plausible groupings are possible depending on the organizing principle; for example, countries could be grouped by geographic continents, cultural regions, economic alliances (e.g., EU vs. non-EU), or linguistic families. The present categorizations therefore represent principled but pragmatic choices intended to provide informative category structure rather than definitive taxonomic claims.

5 This pattern may reflect differences in domain familiarity, where people tend to use a more heterogeneous set of features when they are more familiar with the domain.

6 To aid interpretation of the ICCs, we conducted a simulation study in which similarity judgments were generated from an underlying multidimensional stimulus space. In this simulation, each of 100 of participants based their similarity judgments for *m* = 158 objects pairs (i.e., 5% of the 3160 possible pairs based on 80 objects, the same as the main study design used here) on a subset of 2–5 latent dimensions out of a set of 20 possible latent dimensions, with the degree of overlap in selected dimensions across participants controlled by an “agreement” parameter. Low agreement corresponds to participants relying on largely different subsets of dimensions, whereas high agreement implies that participants attend to similar dimensions when forming similarity judgments. Across 1000 simulations per condition, increasing agreement had little effect on person-level ICCs (approximately .22 across conditions), but substantially increased item-level ICCs (from approximately .13, over .32, to .64, for low, medium, and high agreement between selected dimensions).

7 Importantly, although multidimensional scaling (MDS) and related approaches are often used in the psychological literature to derive dimensions that are interpreted as features representing objects in a psychological space (e.g., Shepard, 1962a; Shepard, 1987; Torgerson, 1952; Medin & Schaffer, 1978), the resulting dimensions should more cautiously be understood as latent coordinates that preserve the average similarity structure and can be used as pragmatic modeling inputs rather than as uniquely identifiable psychological features. That is, MDS primarily performs a form of data reduction, and the correspondence between extracted dimensions and external object properties is not guaranteed and may depend on modeling choices such as the specific MDS variant and the rotation of the solution. Nevertheless, a substantial body of work has demonstrated that similarity-based representations often align meaningfully with interpretable stimulus properties (e.g., Iverson & Kuhl, 1995; Kruskal, 1964; Nosofsky, 1986; Nosofsky et al., 2018c; O’hare, 1976; Shin et al., 1992; Storms et al., 2001) and are consistent with the broader theoretical framework of geometric representations of psychological space, which has also received support from findings in cognitive neuroscience (e.g., Constantinescu, O’Reilly, & Behrens, 2016; Nieh et al., 2021).

8 MDS configurations for each domain with 20 dimensions can be found in the online supplementary materials.

9 Although the optimal MDS solutions included up to 14 dimensions, we restricted visualizations to the first four dimensions for interpretability and comparability across domains. Given the principal-axis rotation, these first four dimensions accounted for a substantial portion of the variance in the dissimilarity data. An approximation of the dimension-wise explained variance in each domain can be found in Appendix C and Table 6

10 To aid interpretation, we collected objective external variables for all stimuli and examined their correlations with the MDS coordinates (see Supplementary Materials). These analyses indicated, for example, that Dimension 3 in the mammals domain was associated with habitat (aquatic vs. terrestrial), whereas Dimension 1 in the country domain correlated with development-related indicators such as life expectancy and the Human Development Index. Importantly, MDS solutions are rotation invariant, meaning that multiple orthogonal transformations yield equivalent representations of the same similarity structure. Consequently, interpretations of individual dimensions depend on the chosen rotation and should be treated as descriptive rather than definitive. It should be noted that interpretation or correspondence of MDS dimensions with external variables can be increased, for instance, by selecting the best interpretable multidimensional scaling rotation based on external variables (Marion, Bibal, & Frénay, 2019) or by imposing external constraints on the configuration as implemented in the smacof package.

11 In the companion paper, we compared five computational cognitive models of estimation with respect to their ability to explain the data of this study. Describing all models and complex fitting algorithms would substantially exceed the scope of the current paper, which focuses on the stimuli and their dimension values.

12 Adjusted $R^2$ is a modified version of the coefficient of determination ($R^2$) that adjusts for the number of predictors in a regression model. While $R^2$ simply measures the proportion of variance in the dependent variable that is explained by the independent variables, it can be artificially inflated by adding more predictors, even if those predictors do not meaningfully contribute to the model. Adjusted $R^2$, in contrast, penalizes model complexity by incorporating the number of predictors relative to the number of observations.

## Code Availability

The scripts to reproduce all analyses reported in the manuscript can be found on the GitHub and OSF repositories of this project.
