# Supplementary material for: Food, mammals, & countries: Stimuli, their pairwise similarities, and MDS dimension values for modeling judgment, categorization, and memory in three real-world domains
Source: Behav Res Methods. 2026 Aug 12;58(9):265. doi: 10.3758/s13428-026-03114-w (PMC13468986; doi:10.3758/s13428-026-03114-w)
Supplement: Supplementary file 1 [file 13428_2026_3114_MOESM1_ESM.pdf]

Supplemental Material:
Food, Mammals, & Countries: Stimuli, their Pairwise Similarities and MDS-Dimension Values for Modeling Judgment, Categorization, and Memory in three Real-World Domains

David Izydorczyk[a] & Arndt Bröder[a]

[a] University of Mannheim

Detail Method of Study 2 - Proof of Concept

Design and Procedure

After providing informed consent, participants were trained in each domain to estimate a specific criterion: the grams of carbohydrates per 100g for food items, life expectancy for countries, or the number of days until female maturity for mammals. During the training phase, participants completed up to 10 blocks of training in which they estimated the criterion values of $k = 12$ exemplars and received immediate feedback about the true values after each response. Exemplars were selected through a simulation procedure aimed at identifying a stimulus set that maximizes discriminability between rule-based and exemplar-based models (the two most widely used and cited estimation processes in the literature) while also covering a broad range of criterion values. Training lasted a minimum of five blocks and continued until participants met an accuracy criterion: at least 10 out of 12 estimates falling within $\pm$ 10% of the true value. Upon reaching this threshold or after completing 10 blocks, participants proceeded to the testing phase, where they estimated the criterion for all 80 stimuli (12 old exemplars and 68 new stimuli) in randomized order without feedback.

Additionally, three attention-check trials were included in the testing phase. In these trials, a basketball was presented instead of a domain-specific stimulus, and participants were instructed to respond with "999". To get an idea about people's prior knowledge of the domain, at the end of the experiment, participants rated their general knowledge of the respective domain on a 7-point scale (1 = very low, 7 = very high). They also answered an open-ended question asking them to describe the strategy they used when making their judgments. In addition, participants provided demographic information and indicated whether their data should be included in the analysis or excluded (Aust et al., 2013). Finally, participants reported whether they had used any form of aid (e.g., note-taking, internet searches), experienced any technical difficulties, and were able to complete the study without

interruption.

The experiment, the full materials, and the preregistration are available at the Open Science Framework (OSF, https://osf.io/4m25d/overview)

Materials

As stimuli for each domain, we used the 80 items presented in the main manuscript, which were selected to capture broad and representative variation within each domain, for instance, foods from different categories (e.g., fruits, vegetables, meat, and sweets), countries from different continental areas, and mammals from a wide range of taxonomic categories.

Participants

All participants were recruited via Prolific. Eligibility criteria required participants to (1) be fluent in English, (2) be at least 18 years old, and (3) have an approval rating above 95%. The study took approximately 30 minutes to complete, and participants were compensated £5. Participants could only participate in one domain.

The planned sample size of approximately $N = 50$ participants per domain was determined based on prior model-based estimation studies, in which samples in the range of 30–60 participants are common (e.g., Hoffmann et al., 2016; Juslin et al., 2003; Pachur & Olsson, 2012). We initially recruited $N = 57$ participants for the food domain, $N = 66$ for the country domain, and $N = 59$ for the mammal domain. As preregistered, participants were excluded if they failed at least one of the three attention checks (excluded: $n = 6/14/9$ for food/country/mammal), indicated that their data should not be used for analysis ($n = 0/2/0$), or reported technical problems ($n = 1/2/0$). No participants were excluded based on the other preregistered criteria: (a) mean response times in the testing phase were more than 3 SDs below the sample mean, (b) participants made only two or fewer distinct estimates for all items in the testing phase, (c) they reported using external aids (e.g., looking up answers, taking notes, or asking others), or (d) they provided judgments in under 1000 ms for more than 10% of the items in the estimation phase. Additionally, we excluded

all participants who answered fewer than 50% of exemplars correctly (i.e., within ±10% of the true value) in the final training block ($n = 4/0/2$). After exclusions, the final sample comprised $N = 46$ participants in the food domain (age: $M = 40.2$, $SD = 13.9$, range = 19 - 68; 67.4% female), $N = 48$ in the country domain (age: $M = 39.7$, $SD = 12.2$, range = 23 - 66; 43.7% female), and $N = 48$ in the mammal domain (age: $M = 37.5$, $SD = 11.4$, range = 21 - 67; 52.1% female).

At the trial level, we excluded trials with unrealistically high estimates in the testing phase, that is, estimates above 100 for the food and country domain and above 10,000 for the mammal domain ($n_t = 6/2/0$). After these exclusions, the final dataset included $N_t = 3,840$ trials in the food domain, $N_t = 3,840$ in the country domain, and $N_t = 3,674$ in the mammal domain.

Correlation of MDS-Dimensions with external variables

To facilitate the interpretability of the extracted multidimensional scaling (MDS) representations, we compiled a set of objective external variables for all stimuli in each domain. These variables are provided alongside the stimulus database in the OSF repository and can be used to relate MDS dimensions to objective environmental properties.

For the food domain, external variables included macronutrient composition (carbohydrates, fat, and protein per 100 g), obtained either from product package descriptions or the German Nutrient Database (Bundeslebensmittelschlüssel; BLS). In addition, we coded categorical food-type variables distinguishing vegetables, fruits, sweets and snacks, spreads, nuts, meat and fish, legumes, dairy products and eggs, and cereal products and potatoes. An additional composition variable grouped fruits and vegetables jointly.

For the country domain, external variables comprised geographic coordinates (latitude and longitude), total carbon dioxide (CO_2) emissions (in metric tons), gross

domestic product (GDP; USD), land area (km^2), population size, population density, and the GINI index (World Bank Database; World Bank, 2026). Further indicators included life evaluation scores (Helliwell et al., 2025), life expectancy, and the Human Development Index (HDI; UNDP, 2025). A categorical variable coded geographic world region or continent membership.

For the mammals domain, variables included average body weight (g), average snout–vent length (cm), average lifespan (years), days until female maturity, and average litter size, extracted from the AnAge database (De Magalhães & Costa, 2009). We additionally included taxonomic classifications and hand-coded categorical variables describing habitat (e.g., aquatic, forest, etc.), diet (e.g., herbivore, carnivore, etc.), and morphological properties (presence of fur, legs, and tail).

To examine relationships between the MDS dimensions and the external variables, we computed correlations between each dimension and all variables (categorical predictors were dummy-coded). The resulting correlation patterns are shown in Figure 1. Exemplary findings include associations between the third mammal dimension and aquatic habitat membership, and between the first country dimension and development-related indicators such as life expectancy and HDI.

Importantly, interpretations of individual MDS dimensions must be treated with caution because MDS solutions are rotation invariant. Any orthogonal rotation of the coordinate system preserves inter-stimulus distances and therefore represents an equivalent solution. Consequently, the apparent psychological meaning of individual dimensions is not uniquely determined but depends on the chosen orientation of the solution in multidimensional space. This rotational indeterminacy is a well-known property of MDS solutions and has motivated recent work proposing methods for selecting rotations that maximize interpretability based on external variables (Marion et al., 2019). Since our primary goal in the main manuscript is to provide psychologically valid similarity

representations that can serve as inputs for cognitive models, rather than to assign fixed semantic interpretations to individual MDS axes, the present analyses should therefore be understood as descriptive aids for interpretation rather than definitive mappings between MDS-dimensions and objective features.

Figure 1

Correlation of MDS-Dimensions with external variables

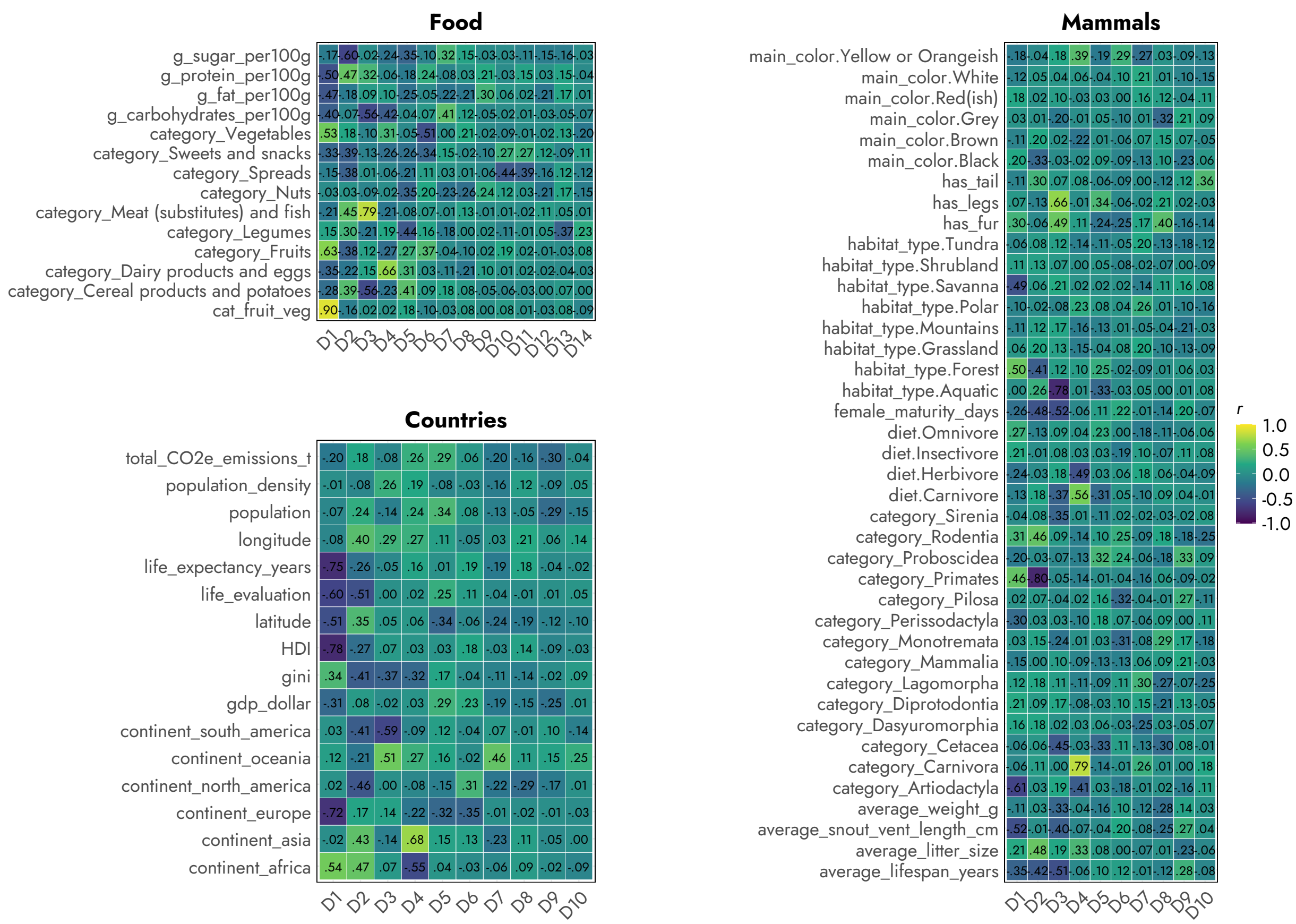

References

Aust, F., Diedenhofen, B., Ullrich, S., & Musch, J. (2013). Seriousness checks are useful to improve data validity in online research. *Behavior Research Methods*, *45*(2), 527–535. https://doi.org/10.3758/s13428-012-0265-2

De Magalhães, J. P., & Costa, J. (2009). A database of vertebrate longevity records and their relation to other life-history traits. *Journal of Evolutionary Biology*, *22*(8), 1770–1774. https://doi.org/10.1111/j.1420-9101.2009.01783.x

Helliwell, J. F., Layard, R., Sachs, J. D., De Neve, J.-E., Aknin, L. B., & Wang, S. (Eds.). (2025). *World happiness report 2025*. Wellbeing Research Centre, University of Oxford.

Hoffmann, J. A., von Helversen, B., & Rieskamp, J. (2016). Similar task features shape judgment and categorization processes. *Journal of Experimental Psychology: Learning, Memory, and Cognition*, *42*(8), 1193–1217. https://doi.org/10.1037/xlm0000241

Juslin, P., Olsson, H., & Olsson, A. C. (2003). Exemplar effects in categorization and multiple-cue judgment. *Journal of Experimental Psychology*, *132*(1), 133–156. https://doi.org/10.1037/0096-3445.132.1.133

Marion, R., Bibal, A., & Frénay, B. (2019). BIR: A method for selecting the best interpretable multidimensional scaling rotation using external variables. *Neurocomputing*, *342*, 83–96. https://doi.org/10.1016/j.neucom.2018.11.093

Pachur, T., & Olsson, H. (2012). Type of learning task impacts performance and strategy selection in decision making. *Cognitive Psychology*, *65*(2), 207–240. https://doi.org/10.1016/j.cogpsych.2012.03.003

UNDP. (2025). *Human development index (hdi)*. United Nations Development Programme. Retrieved April 1, 2026, from https://hdr.undp.org/data-center/human-development-index#/indicies/HDI

World Bank. (2026). *World bank open data*. Retrieved April 1, 2026, from https://data.worldbank.org/
